# Design, Characterization, and Evaluation of scFvCD133/rGelonin: A CD133-Targeting Recombinant Immunotoxin for Use in Combination with Photochemical Internalization

**DOI:** 10.3390/jcm9010068

**Published:** 2019-12-26

**Authors:** Cathrine Elisabeth Olsen, Lawrence H. Cheung, Anette Weyergang, Kristian Berg, Daniel A. Vallera, Michael G. Rosenblum, Pål Kristian Selbo

**Affiliations:** 1 Department of Radiation Biology, Institute for Cancer Research, Oslo University Hospital, The Norwegian Radium Hospital, N-0310 Oslo, Norway; cathrieo@gmail.com (C.E.O.); Anette.Weyergang@rr-research.no (A.W.); Kristian.Berg@rr-research.no (K.B.); 2 Department of Experimental Therapeutics, The University of Texas MD Anderson Cancer Center, Houston, TX 77030, USA; lcheung@mdanderson.org (L.H.C.); mrosenbl@mdanderson.org (M.G.R.); 3 Department of Therapeutic Radiology-Radiation Oncology, University of Minnesota, Masonic Cancer Center, Minneapolis, MN 55455, USA; valle001@umn.edu

**Keywords:** cancer stem cells, CD133, prominin-1, photochemical internalization, photodynamic therapy, immunotoxins, ribosome-inactivating proteins, drug delivery

## Abstract

The objective of this study was to develop and explore a novel CD133-targeting immunotoxin (IT) for use in combination with the endosomal escape method photochemical internalization (PCI). scFvCD133/rGelonin was recombinantly constructed by fusing a gene (scFvCD133) encoding the scFv that targets both non-glycosylated and glycosylated forms of both human and murine CD133/prominin-1 to a gene encoding the ribosome-inactivating protein (RIP) gelonin (rGelonin). RIP-activity was assessed in a cell-free translation assay. Selective binding and intracellular accumulation of scFvCD133/rGelonin was evaluated by flow cytometry and fluorescence microscopy. PCI of scFvCD133/rGelonin was explored in CD133^high^ and CD133^low^ cell lines and a CD133^neg^ cell line, where cytotoxicity was evaluated by the MTT assay. scFvCD133/rGelonin exhibited superior binding to and a higher accumulation in CD133^high^ cells compared to CD133^low^ cells. No cytotoxic responses were detected in either CD133^high^ or CD133^low^ cells after 72 h incubation with <100 nM scFvCD133/rGelonin. Despite a severe loss in RIP-activity of scFvCD133/rGelonin compared to free rGelonin, PCI of scFvCD133/rGelonin induced log-fold reduction of viability compared to PCI of rGelonin. Strikingly, PCI of scFvCD133/rGelonin exceeded the cytotoxicity of PCI of rGelonin also in CD133^low^ cells. In conclusion, PCI promotes strong cytotoxic activity of the per se non-toxic scFvCD133/rGelonin in both CD133^high^ and CD133^low^ cancer cells.

## 1. Introduction

Cancer stem cells (CSC), or tumor initiating cells, are highly aggressive malignant cells that have gained stem cell biology including capacity to both self-renew and differentiate, hence making them responsible for maintaining tumor heterogeneity [1,2]. In addition, CSCs are more therapy resistant than differentiated cancer cells, and for that reason are suggested to be the drivers of tumor relapse after therapy [3,4,5,6]. However, as differentiated cancer cells can dedifferentiate and gain stem cell biology after hypoxia-mediated changes in the tumor microenvironment (TME) [7] or as a response to therapy-mediated alteration of the TME [1], it is important to establish new therapeutic interventions that simultaneously target and kill both CSCs and differentiated cancer cells [8]. Such strategies may involve multimodality treatments including immunotoxins (IT) targeting e.g., CD133-expressing [9,10] or LGR5+ [11] CSCs.

CD133 (prominin-1) is a pentaspan glycoprotein over-expressed on several types of CSCs and is an independent prognostic factor for clinical outcome in patients with malignant glioma and colon and breast carcinomas [12,13,14,15]. CD133 is suggested to be involved in therapy resistance and metastasis [16,17,18,19] and tumor vascularization [20]. Hence, CD133 has gained increasing interest as a drug target for eradication of aggressive tumors [21]. Since CD133 is also expressed in normal hematopoietic stem cells and neuronal and endothelial progenitors, targeting of CD133 bears the risk of affecting healthy tissue. In spite of this, only modest cytotoxic effects on murine normal progenitor cells have been reported after systemic administration of CD133-targeting immunotoxins, which was related to lower CD133 expression levels in normal progenitor cells compared to CSCs [21]. Furthermore, immunological targeting of CD133 by a vaccine, ICT-121, containing autologous dendritic cells loaded with two HLA-A2 restricted epitopes of the CD133 antigen, in patient with recurrent gliomas was found both safe and well-tolerable [22]. However, as putative CSC-markers—including CD133—are expressed on normal stem or progenitor cells, strategies that enhance the specificity and efficacy of CSC-targeting immunotoxins are warranted to avoid potential adverse effects.

Photochemical internalization (PCI) is a light-controlled cytosolic delivery method for drugs trapped in endocytic vesicles [23,24] that is based on the same principles of photodynamic therapy (PDT) [25]. PCI is a combinatorial application of the clinical relevant amphiphilic photosensitizer TPCS_2a_ (fimaporfin) [26], which after co-administration with a drug (e.g., a macromolecule such as an immunotoxin or a small drug/chemo) accumulate and co-localize in endosomes and lysosomes in cancer cells [10,24]. Light activation of TPCS_2a_ results in excessive generation of reactive oxygen species (ROS) of which singlet oxygen (^1^O_2_) is the most common ROS [27]. Due to the high synergy between the components of the PCI strategy, the technology widens the therapeutic window of drugs entrapped in endosomes and lysosomes and, therefore, holds the potential of reducing the risk of adverse events of drugs, including immunotoxins [28]. The PCI-photosensitizer TPCS_2a_ is not a substrate of the plasma membrane pumps ABCG2/BCRP1 [29] and P-glycoprotein (ABCB1) [30,31], hence making the PCI technology suitable for targeting of CSCs. Recently, we demonstrated the proof-of-concept of PCI of a CD133-targeting immunotoxins both in vitro and in vivo using either the biotinylated CD133-targeting mAb AC133 bound to the ribosome-inactivating protein toxin Saporin-streptavidin (a complex of >700 kDa) [32] or the chemical IT conjugate AC133-saporin (244 kDa) [10,33]. Despite the very high potent activity of PCI of AC133-saporin in vitro, and the accumulation of AC133-saporin in tumor tissue, we only obtained modest anti-tumor activity after PCI. One reason for this could be its relatively large molecular size and resultant reduced tumor penetration capacity.

In the present work, we aimed to develop a small and novel, recombinant anti human and mouse CD133-targeting immunotoxin for use in combination with the endosomal escape drug delivery technology PCI. We designed and recombinantly produced a 59 kDa fusion construct consisting of the scFv fragment of the anti-CD133 mAb (clone CD133 HB#7 also named hybridoma clone 7) fused together with the ribosome-inactivating protein toxin gelonin (rGelonin) making the immunotoxin scFvCD133/rGelonin. scFvCD133/rGelonin was evaluated for specific cellular binding, uptake and in vitro cytotoxicity with or without PCI. Cell lines with, according to the literature, negative (NIH/3T3), low (U87, MCF-7 and MDA-MB-231) and high (WiDr and HT29) CD133 expression were included in this study.

## 2. Experimental Section

### 2.1. Photosensitizer and Chemical

The PCI photosensitizer tetraphenyl chlorin disulphonate (TPCS_2a_/fimaporfin, kindly provided by PCI Biotech AS, Oslo, Norway) was dissolved at 0.4 mg/mL in 3% Tween 80, 2.8% mannitol, 50 mM Tris, pH 8.5 (all from Sigma-Aldrich, Currently Merck, Darmstadt, Germany), and kept protected from light at 4 °C. All other chemicals were, if not otherwise stated, purchased from Sigma-Aldrich.

### 2.2. Design and Recombinant Production of scFvCD133/rGelonin

**Figure 1:**
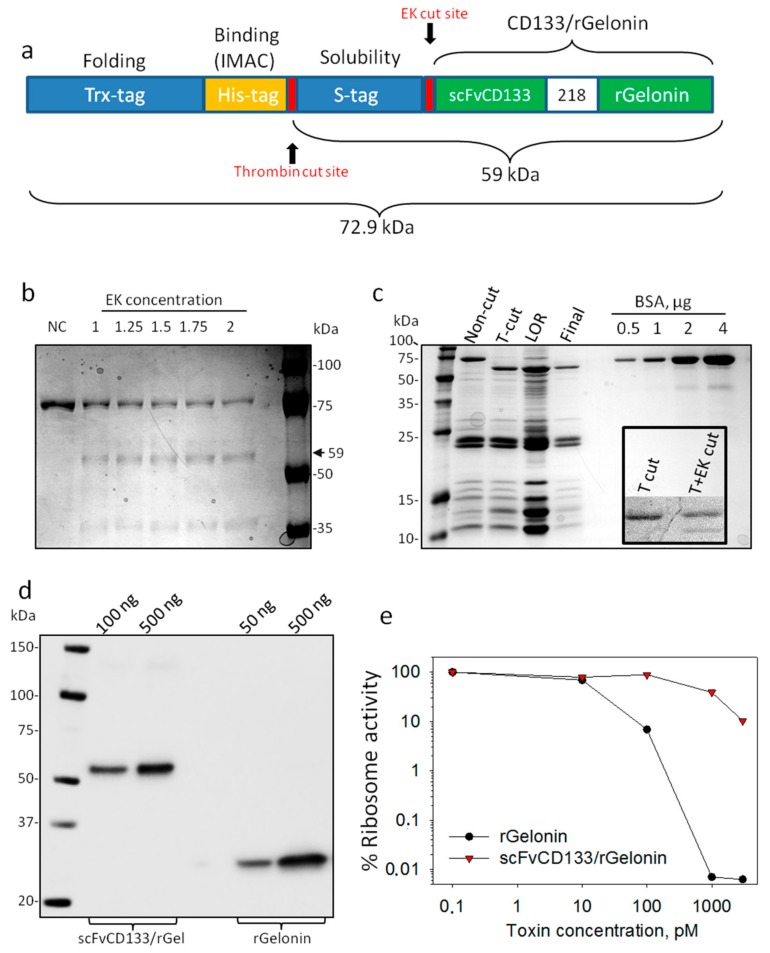
Design, expression and RIP activity of scFvCD133/rGelonin. (**a**) Schematic representation of the recombinant scFvCD133/rGelonin fusion construct including the enterokinase (EK) and thrombin cut sites. (**b**) Coomassie Blue stain of non-cut (NC) and EK cut material with increasing concentration of enterokinase. (**c**) Coomassie Blue stain of non-cut and thrombin cut (T-cut) material, the material left on resin (LOR) upon His-tag removal (concentrated), and the final cut and purified product (Final). Increasing concentrations of bovine serum albumin (BSA) were used as control. Inserted panel under the BSA bands shows an attempt on cutting the final product with EK upon T-cut. (**d**) Western blot immunodetection of scFvCD133/rGelonin and rGelonin. The immunoblot is representative of three individual experiments with different toxin concentrations. (**e**) Ribosome-inactivating protein activity of scFvCD133/rGelonin and rGelonin measured by using the cell free Rabbit reticulocyte lysate assay. Representative single well-based data from three independent experiments.

The cDNA encoding human CD133 scFv (anti-CD133 hybridoma clone 7) and recombinant gelonin were fused together by using the splice overlap extension PCR (OE-PCR) method [34] with CD133 and recombinant gelonin DNA as templates. To construct scFvCD133-rGelonin, an upstream OE-PCR fragment encoding the restriction enzymes BspHI was amplified from the N-terminal portion of the scFvCD133 gene using the oligonuceotide primers BSPH-CD133 For(5′GGCTCCGTCATGACAGACATTGTTCTCTCCCAG-3′), and Link CD133 Bac Rev(5′-ACTACCCTCACCACTCCCGGGCTTACCACTGCCGCTGGTGGAGCCTGAGGAGACTGTGAGA GT-3′). An adjoining downstream OE-PCR fragment encoding a 218 flexible linker (GSTSG SGKPGSGEGSTKG) and a restriction enzyme HINDIII was amplified from the recombinant deglycosylated gelonin gene using the oligonucleotide primers Link rGel For(5′-GGTAAGCC CGGGAGTGGTGAGGGTAGTACTAAGGGTGGTCTAGACACCGTGAGC-3′), and rGel Bac Rev(5′-GGAGCCACCAAGCTTATCACTATTTAGGATCTTTATCGACGAA-3′). The upstream and downstream OE-PCR fragments were then reassembled as a full-length fusion scFvCD133-rGelonin gene encoding the fusion protein by an additional PCR step using a pair of oligonucleotide primers BSPH-CD133 For and rGel Bac Rev flanking the 5′-and 3′-end (see above). The final PCR fragment was purified and cleaved with BspHI and HindIII restriction endonucleases and then cloned into the Novagen pET-32a expression vector that utilizes the T7 promoter for the transcriptional control of the inserted fusion gene between the NcoI and HindIII site. The fusion scFvCD133-rGelonin gene construct was verified by DNA sequencing before protein expression. The constructs were then transformed into NEB Escherichia coli strain T7 Express Iq and SoluBL21 Competent for expression of the fusion protein. The final product consisted of deglycosylated recombinant gelonin (28.5 kDa vs. 30 kDa for natural gelonin) and scFvCD133 recombinantly fused by the 218 flexible peptide linker (GSTSGSGK PGSGEGSTKG). The scFvCD133/rGelonin construct was designed with a Trx-and S-tag for the correct protein folding and solubilization in addition to a His-tag for purification by IMAC (totally 72.9 kDa). Two cutting sites (the enterokinase (EK) and thrombin cut sites) were provided on the construct for purification, as shown in Figure 1a.

Induction of bacteria: T7 Express Iq and SoluBL21 Competent E. Coli were grown in autoclaved LB media or M9 media supplemented with 0.1 mM calcium chloride, 1 mM magnesium sulfate, 0.3% glycerol and 200 µg/mL Ampicillin, at 37 °C, respectively. Protein expression was induced by adding 1 mM isopropyl 1-thio-beta-d-galactopyranoside (IPTG) at 25 °C for 5 h. Induced bacterial cultures were pelleted (5000 rpm, 15 min, 4 °C) and stored at −20 °C until use. Thawed pellet was resuspended in 800 mL 20 mM Tris (pH 8.0) and 300 mM NaCl, and lysed by microfluidization on ice. The bacterial lysate was then ultracentrifuged (40,000 rpm, 30 min, 4 °C).

Immobilized metal ion affinity chromatography (IMAC): The lysate was loaded onto a Co^2+^-containing column (Talon) for purification. The column was washed with 200 mL 20 mM Tris (pH 8), 300 mM NaCl and 5 mM imidazole, and fusion toxin eluted with 5–10 mL 20 mM Tris (pH 8.0), 250 mM NaCl and 1 M imidazole.

Dialysis and cutting: The fusion toxin was dialyzed into 20 mM Tris (pH 7.4–7.8) and 150 mM NaCl and digested for 3–5 h with thrombin to cut off the His-tag. Then the salt concentration was adjusted to 350 mM NaCl and 10 mM imidazole before reloaded on a Co^2+^-containing column (Talon) to remove the His-tag. The flow-through (fusion toxin) was dialyzed overnight into 300 mM phosphate-buffered NaCl and frozen at −20 °C. The immunotoxin was sterile filtered using a 0.22 µm sterile filter connected to a syringe. The protein concentration of scFvCD133/rGelonin was detetced by the DC Protein assay (BioRad, Hercules, CA, USA) using rGelonin as control, and BSA as standard.

### 2.3. Coomassie Blue Stain

Immunotoxin and BSA controls (Sigma-Aldrich, St Louis, MO, USA) were diluted in 5× Reducing Sample Buffer (250 mM Tris-HCl, pH 6.8, 10% SDS, 30% glycerol, 5% β-mercaptoethanol, 0.02% bromophenol blue) and applied on 12% polyacrylamide gels together with Perfect Protein Marker (Novagen). Sodium dodecyl sulfate polyacrylamide gel electrophoresis (SDS-PAGE) was ran at 160–180 V for 20 min. Then gels were activated in destain buffer (40% methanol, 10% glacial acetic acid) before staining of proteins in a 0.1% Coomassie brilliant blue solution. Destaining was performed in destain buffer gradually diluted with water.

### 2.4. Western Blot Assay

Immunotoxin and rGelonin control were diluted in PBS and further diluted in 5× Reducing Sample Buffer as described above, and applied on 4–20% TGX gels (BioRad). SDS-PAGE was ran at 160–180 V for 30 min, and proteins were subsequently transferred onto PVDF membranes by the Trans Blot Turbo method as recommended by the producer (Bio-Rad, Hercules, CA, USA). The membrane was blocked in 5% milk for 1 h at room temperature before overnight incubation with a polyclonal rabbit anti-rGelonin antibody (either from Rosenblum lab or our lab, both works equally) at 4 °C. After 3× wash in TTBS (50 mM Tris-HCl, 150 mM NaCl, pH 7.6, 0.05% tween 20), the membrane was incubated with anti-rabbit-HRP. LumiGlo or Super Signal Dura was used for signal development by the ChemiDoc system as recommended by the producer (Bio-Rad).

### 2.5. Cell Culture

Human colon adenocarcinomas WiDr (ATCC CCL-218) and HT29 (ATCC HTB-38), human breast cancer cell lines MDA-MB-231 (ATCC HTB-26) and MCF7 (ATCC HTB-22), murine fibroblastoma NIH/3T3 (ATCC CRL-1658) and human glioblastoma U87 (ATCC HTB-14) were obtained from the American Type Culture Collection (LGC Standards AB, Boras, Sweden). The cells were grown at 37 °C, 5% CO_2_ in media as recommended by ATCC, supplemented with 10% FBS, 100 IU/mL penicillin and 100 µg/mL streptomycin (Sigma-Aldrich) and sub-cultivated every third day. Work with live cells was performed in a laminar airflow hood. All cell lines were tested for mycoplasma by the MycoAlert kit (Lonza, Basel, Switzerland) and proven mycoplasma negative.

### 2.6. Flow Cytometry

Cells trypsinated and harvested for detection of CD133 were washed and blocked with 0.5% BSA in PBS for 10 min on ice. Cells were resuspended in 0.5% BSA in PBS and incubated 1 h on ice with 12.3 µg/mL mouse anti-human CD133/Prominin-1 mAb (DSHB Hybridoma Product clone: CD133 HB#7, deposited by Swaminathan, S.K., Panyam, J. and Ohlfest, J.R. [35]. Cells were then washed in 0.5% BSA, and resuspended in rabbit anti-mouse-FITC Dako antibody (Agilent, Santa Clara, CA, USA) (1:50), and further incubated 30 min on ice. Cells were subsequently washed once in 0.5% BSA and then resuspended in PBS. FITC was excited by a 488 nm (50 mW) laser, and emission detected by a 525/50 nm band pass filter combined with a 505 nm longpass filter. Fluorescence analyses were performed in LSRII flow cytometers (Becton-Dickinson, San Jose, CA, USA). To assess if TPCS_2a_ was blocking the CD133 binding, cells were preincubated overnight with 0.2 µg/mL TPCS_2a_ before antibody incubation as described above. Cells with TPCS2a were gated based on TPCS_2a_ excitation by a 407 nm (100 mW) laser, and emission detected by a 660/20 nm band pass filter combined with a 635 nm longpass filter.

Cells harvested for intracellular detection of CD133 were permeabilized by drop-wise methanol fixation. After one wash in PBS the cells were stained with 12.3 µg/mL HB#7 anti-CD133 in 5% nonfat milk (In 1× TBS. Centrifuge with 2000× *g* for 1 min to remove aggregates before use) for 30 min at room temperature, before 2× wash with PBS and subsequent incubation with 1:50 dilution of rabbit anti-mouse-FITC in 5% milk for 30 min in room temperature. Cells were washed once before analysis by flow cytometry. Anti-mouse-FITC was excited and detected as described above.

Detection of cellular scFvCD133/rGelonin by using an anti-gelonin polyclonal antibody: HT29 and U87 cells were seeded out at 200,000 cells/well in 6 well plates, and incubated with 10 nM rGelonin or scFvCD133/rGelonin for 20 h. Then cells were trypsinated, transferred to flow tubes, washed with PBS and fixed with methanol. Control cells were stained using 0.05 µM CellTrace Violet (Molecular Probes) prior to methanol fixation. The stained control cells were added to the samples prior to antibody incubation. After methanol fixation, cells were washed once in PBS, and incubated with a polyclonal rabbit anti-rGelonin (from Rosenblum lab or our lab) (1:500, 50 µL 5% milk) for 45 min at room temperature. Then the cells were washed twice in PBS and incubated with goat anti-rabbit-Alexa488 secondary antibody (Invitrogen, Carlsbad, CA, USA) (1:200, 50 µL 5% milk) for 45 min at room temperature. Cells were then washed twice in PBS and analyzed by flow cytometry based on SSC area and width (single cells). CellTrace Violet was excited by a 407 nm (100 mW) laser, and emission detected by a 450/50 nm band pass filter. Alexa488 was excited by a 488 nm (50 mW) laser, and emission detected by a 525/50 nm band pass filter combined with a 505 nm longpass filter.

Fluorescence-activated cell sorting (FACS) of CD133^high^ and CD133^low^ HT-29 cells: Thirty million cells were stained with 12.3 µg/mL anti-CD133 mAb HB#7 in 500 µl 0.5% BSA for 30 min at 37 °C. Then cells were washed once with 0.5% BSA, and further stained with 1:50 rabbit-anti-mouse-FITC in 0.5% BSA for 15 min at 37 °C. The cells were washed again and resuspended in 1 mL McCoy’s 5a containing 1 mM EDTA. Cells with the 5% highest and 5% lowest signal and the bulk were sorted into 50% conditioned McCoy’s 5a medium, providing metabolites and growth factors to enhance survival of the FACS cells, using an Aria IIu SORP high speed sorter, and seeded for sensitivity experiment (MTT) after media replacement. Propidium iodide (PI, Sigma-Aldrich) staining was included in all staining protocols to identify and exclude dead cells. 1 µL PI (final PI concentration: 3 µM) was added directly into each flow tube 10 min before flow cytometry or FACS.

### 2.7. Live Cell Fluorescence Microscopy

The CD133^high^ HT29 and the CD133^low^ U87 cell line were chosen for fluorescence microscopy of scFvCD133/rGelonin and rGelonin binding and internalization after incubation with 10 nM toxin for 1–2 h. Then cells were methanol fixed and incubated with anti-rGelonin (rabbit) (1:500) for 30–60 min, followed by goat anti-rabbit-PE (1:50) (Invitrogen) or anti-rabbit-Alexa488 (1:200) for 30 min. The CD133^high^ HT29 and the CD133^low^ MDA-MB-231 cell lines were chosen for evaluation of cellular binding and uptake of the CD133-targeting mAb anti-CD133 (HB#7, having the same binding moiety on the CD133 receptor as the scFVCD133) by means of fluorescence microscopy. Fifty thousand cells were seeded on coverslips in 4-well Nunc plates (Invitrogen). After 1 h and 24 h of incubation with 10 nM mouse anti-CD133 mAb, the cells were methanol fixed, and further incubated with anti-mouse-FITC (1:50) for another 30 min.

The coverslips were flipped onto microscopy slides on a droplet of 40% glycerol in PBS. Cells were imaged by a Zeiss Axioplan epi-fluorescence and phase-contrast microscope using 63× magnification with an oil immersion objective (Carl Zeiss AG, Oberkochen, Germany). The fluorescence was detected with an AxioCamMR3 camera (Carl Zeiss). FITC and Alexa488 fluorescence was recorded using a 470/40 nm band pass excitation filter, a 495 nm dichroic mirror, and a 525/50 nm long pass emission filter. For PE a 550/25 nm band pass excitation filter, a 570 nm dichroic mirror, and a 605/70 nm band pass emission filter were used. The software program Axio Vision Analysis (Carl Zeiss) was used to process and analyze the digital micrographs.

### 2.8. Rabbit Reticulocyte Lysate Translation Assay Protein Synthesis

Evaluation of ribosome-inactivating protein (RIP) catalytic activity of scFvCD133/rGelonin and rGelonin was determined by using the cell free Rabbit Reticulocyte Lysate assay (Promega) as previously described [36].

### 2.9. Evaluation of Therapy-Induced Cytotoxic Responses

Cytotoxicity was measured by the MTT viability assay 72 h after incubation start or light exposure (PCI). For efficacy evaluation of the toxins (rGelonin and CD133ScFv/rGelonin), WiDr (1500 cells/well), HT29 (2000 cells/well), U87 (2000 cells/well), NIH/3T3 (750 cells/well), MDA-MB-231 (6000 cells/well), and MCF7 (2000 cells/well) were seeded in 96-well plates (Nunc) and left for overnight attachment. Then cells were incubated with increasing concentrations of immunotoxin and rGelonin standard for 72 h before incubation with 0.4 mg/mL MTT for 4 h. Formazan-crystals were solubilized in DMSO, and absorbance read at 570 nm.

For the PCI protocol cells were seeded as described above, and incubated for 18 h with 0.4 µg/mL TPCS_2a_. Then the cells were washed twice with PBS (with Mg^2+^ and Ca^2+^) and incubated with scFvCD133/rGelonin or rGelonin for 2–4 h, before illumination with LumiSource^®^ (PCI Biotech AS, Lysaker, Norway). Cell viability was measured as described above 72–96 h post light exposure. The lamp consists of four 18-W Osram L 18/67 light tubes and delivers blue light (λ_max_ = 435 nm) with an iradiance of 13.5 mW/cm^2^. The irradiance of the lamp varies <10% across the illumination area (45 × 17 cm).

### 2.10. Statistics

Sigmaplot version 14.0 (Systat Sofware Inc., San Jose, CA, USA) was used for statistical analysis where *p* ≤ 0.05 was considered statistically significant. Two-sided Student’s *t*-test was performed.

## 3. Results

### 3.1. Production, Purification, and Evaluation of Ribosome Inactivating Activity of scFvCD133/rGelonin

The scFvCD133/rGelonin fusion construct (Figure 1a) was expressed in T7 Express Iq and SoluBl21 and secreted into the culture media. Following purification by IMAC a protein with the correct estimated size of 72.9 kDa was detected on Coomassie blue stained polyacrylamide gels (Figure 1b NC). Enterokinase did not cleave the protein sufficiently (Figure 1b), thrombin digestion was, however, found much more efficient and resulted in a 59 kDa product (still containing the S-tag). A final product of high purity was confirmed upon His-tag removal by IMAC (Figure 1c,d). The scFvCD133/rGelonin concentration was measured to be 1.65 µM based on Lowry assay (data not shown). This final product was, however, also unsuccessfully cut by enterokinase. Approximately 1200 µg S-tagged scFvCD133/rGelonin was obtained per 10 L bacteria.

Gelonin is a RIP class I protein, which exerts N-glycosidase activity on the 28S rRNA unit of ribosomes by cleaving out adenine at the 4324 site. scFvCD133/rGelonin reduced ribosomal activity with increasing immunotoxin concentration. Compared to the activity of rGelonin, scFvCD133/rGelonin had, however, lost its activity ~100-fold as measured at IC90 (Figure 1e).

### 3.2. Detection of CD133 Expression by Flow Cytometry and Fluorescence Microscopy

**Figure 2:**
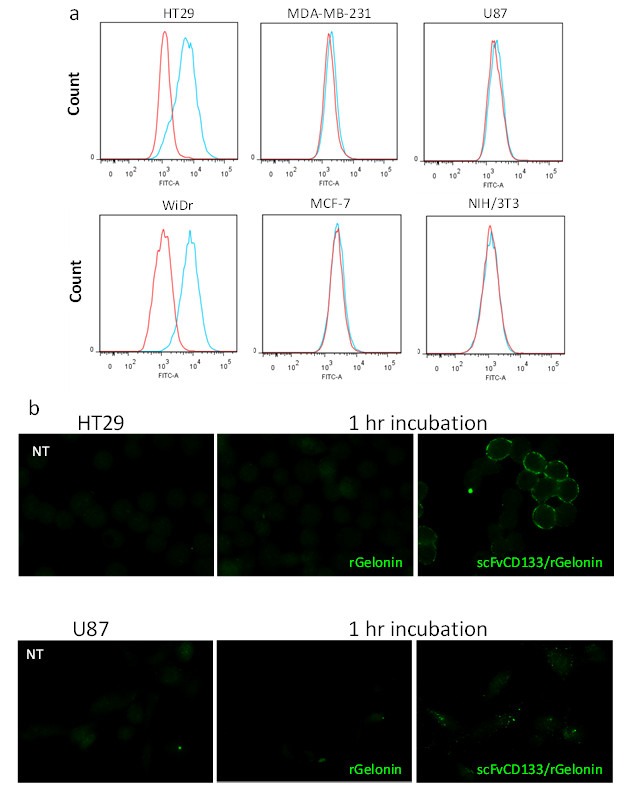
Characterization of CD133 plasmamembrane expression and binding/internalization of scFvCD133/rGel. (**a**) Detection of CD133 expression in live cells assessed by flow cytometry after 1 h. ice-cold incubation of the mouse primary antibody anti-CD133 (HB#7, which have the same binding site on the CD133 receptor as the scFV-fragment of the scFvCD133/rGel immunotoxin) and a secondary anti mouse-FITC antibody. The result is representative from three individual experiments, and in addition to three other experiments performed in methanol fixed cells. (**b**) Fluorescence microscopy 1 h. after incubation at 37 °C with either 10 nM rGelonin or scFvCD133/rGelonin in HT29 and U87 cells (as indicated in the panels). Cells were fixed with methanol, and both toxin (rGel) and immunotoxin (scFvCD133/rGel) was detected by a polyclonal anti-rGelonin antibody and a secondary anti-rabbit mAb-Alexa488. The micrograph is a representative result from three individual experiments.

In our experiments, the CD133 antibody (clone: CD133 HB#7) from the same clone as our recombinant immunotoxin was used for CD133 identification. We first determined the expression of CD133 on the human colon adenocarcinomas HT29 and WiDr, the human breast cancer cell lines MDA-MB-231 and MCF7, the human glioblastoma U87 and the murine embryonic fibroblasts NIH/3T3 by flow cytometry. Following on-ice incubation with anti-CD133 and anti-mouse-FITC in live cells, the CD133 receptor was found over-expressed on the plasma membrane of the HT29 and WiDr cell lines, while not in MDA-MB-231, MCF7, U87, and NIH/3T3 cells (Figure 2a).

scFvCD133/rGelonin was by fluorescence microscopy found bound to the plasma membrane, and to a some degree as intracellular, granular puncta in the CD133^high^ HT29 cells after 1 h incubation, while no membrane binding of scFvCD133/rGelonin was detected on the CD133^low^ U87 cells 1 h after incubation with scFvCD133/rGelonin. However, intracellular fluorescence puncta from scFvCD133/rGelonin was detected in the U87 cells (Figure 2b). Fluorescence microscopy also revealed binding of the CD133 receptor-targeting mAb (Clone CD133 HB#7) on the plasma membrane of the CD133^high^ HT29 cell line after 1 h incubation, while no binding after 1 h incubation was detected in the CD133^low^ MDA-MB-231 cell line (Supplementary Figure S1). At 24 h incubation, intracellular fluorescence puncta of the anti-CD133 mAb was observed in HT29 cells, while very low intracellular fluorescence was detected in the MDA-MB-231 cells (Supplementary Figure S1).

### 3.3. In Vitro Cytotoxicity of Free rGelonin versus scFvCD133/rGelonin without PCI

**Figure 3:**
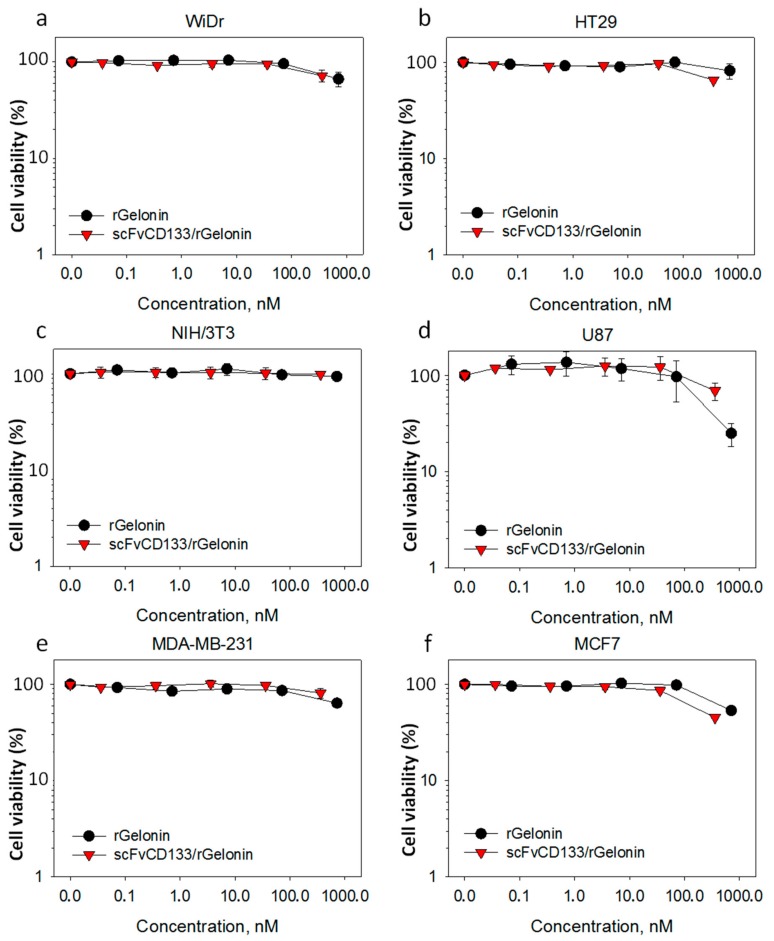
Cytotoxic effects of free rGelonin versus scFvCD133/rGelonin without PCI. Cytotoxicity was assessed in six different cell lines, including WiDr (**a**), HT29 (**b**), NIH/3T3 (**c**), U87 (**d**), MDA-MB-231 (**e**), and MCF-7 (**f**) by the MTT viability assay 72 h after incubation with increasing concentrations of rGelonin or scFvCD133/rGelonin. The data are the mean of two individual experiments. Some of the error bars are smaller than the symbol size. Error bars = S.E.

For all 6 cell lines, no cytotoxic responses were induced by either scFvCD133/rGelonin or rGelonin at concentrations ≤100 nM (Figure 3a–f). At concentrations >100 nM, the viability dropped slightly in all cell lines except for the CD133^neg^ cell line NIH/3T3 cells (Figure 3c). Surprisingly, the CD133^low^ U87 cell line exhibited the highest sensitivity to rGelonin (Figure 3d). Using higher concentrations than 333 nM scFvCD133/rGelonin were limited by the stock concentration of the fusion toxin.

### 3.4. Cytotoxic Responses after PCI of scFvCD133/rGelonin Is Independent on the Degree of CD133 Expression

**Figure 4:**
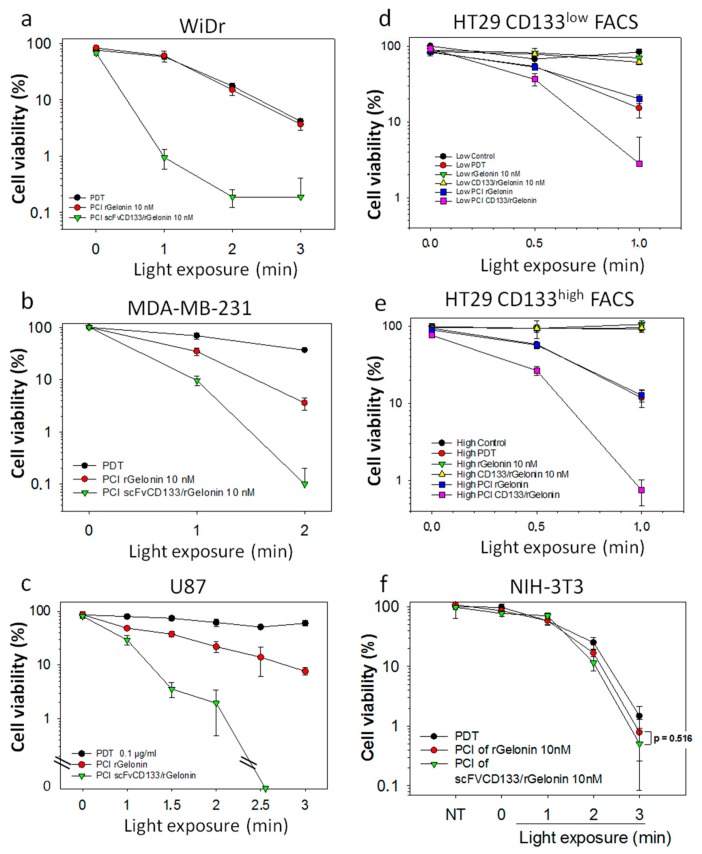
Light dose-dependent cytotoxic effects of PCI of rGelonin versus PCI of scFvCD133/rGelonin in cells with various expression of CD133. WiDr ((**a**), CD133^high^), MDA-MB-231 ((**b**), CD133^low^), U87 ((**c**), CD133^low^), HT-29 ((**d**,**e**), CD133^high^), and NIH-3T3 ((**f**), CD133^neg^). Incubation set-up described in Section 2.9. The data are the mean of triplets within the experiments, which were reproduced at least twice. CD133 high (**d**) and low (**e**) sorted HT29 cells subjected to PCI of 10 nM rGelonin and scFvCD133/rGelonin incubated for 2 h (Cntrl = untreated cells, PDT = TPCS_2a_ in combination with light, rGel = rGelonin). The data in (**f**) is representative of two independent experiments, while (**a**–**e**) data are representative of at least three individual experiments. Error bars = S.D.

PCI of 10 nM scFvCD133/rGelonin induced very high cytotoxic responses compared to PCI of free rGelonin in all 6 cell lines; WiDr (Figure 4a), MDA-MB-231 (Figure 4b), U87 (Figure 4c) and HT-29 (Figure 4d,e). To study if the sensitivity was dependent on the CD133 expression level, the 5% top (Figure 4d) and low (Figure 4e) CD133 expressing HT29 cells were FACSorted and subsequently treated with PCI. At 1 min light dose, the PCI of scFvCD133/rGelonin-mediated cytotoxicity was found 8-fold higher than that of PCI of rGelonin in the CD133^low^ FACSorted cells (Figure 4d) and 30-fold higher than that of PCI of rGelonin in the CD133^high^ FACSorted cells (Figure 4e). At 1 min light exposure, there were no significant differences between PDT and PCI of rGelonin in both CD133^low^ and CD133^high^ cells, which is in line with the observations in the WiDr cells (Figure 4a).

Altogether, these data suggest a specificity of scFvCD133/rGelonin towards the CD133 receptor. PCI of scFvCD133/rGelonin induced high cytotoxic responses compared to PCI of rGelonin in both CD133^high^ and CD133^low^ cell lines. However, the efficacy of PCI of scFvCD133/rGelonin is independent on the level of plasma membrane expression of CD133. Thus, the high PCI-induced cytotoxic effects in CD133^low^ cells are of concern as this may affect surrounding normal CD133-expressing cells. However, an advantage of the PCI technology is the spatiotemporal drug activation controlled by the light-directed exposure of the tumor only.

## 4. Discussion

The aim of the present work was to develop a small CD133-targeting immunotoxin (scFvCD133/rGel), by recombinantly fusing the highly specific scFvCD133 (clone: CD133 HB#7), recognizing both human and murine glycosylated and non-glycosylated extracellular domains of CD133, to the type I RIP gelonin. The targeting of both human and murine CD133/prominin-1 will give us future possibilities to evaluate this construct in both immunocompetent (syngeneic models) and immunodeficient (xenograft models) mice strains. Since scFvCD133/rGelonin also is independent of the glycosylation status of the receptor we may bypass potential problems with detecting different post-translational versions of CD133/prominin-1 [37]. Gelonin is rigidly packed and relatively inaccessible to proteolytic cleavage [38], making the toxin favorable for construction of recombinant immunotoxins for use in combination with the endosomal/lysosomal escape technology PCI. Gelonin is additionally reported to be 5–10 times less toxic to whole cells than saporin [39], making rGelonin an even more useful agent for potential clinical use in terms of reducing adverse effects. This study is the first report on the development and production of a CD133-targeting recombinant fusion toxin aimed for the specific delivery by PCI. The obtained recombinant IT size of 59 kDa should be sufficiently small for adequate tumor penetration [40] but yet probably large enough to prevent substantial loss through renal clearance [41]. The purification process after bacterial production resulted in scFvCD133/rGelonin with high purity. An additional step of size exclusion chromatography should, however, be applied to remove impurities <35 kDa. The RLLA assay showed a dramatic reduction of scFvCD133/rGelonin RIP activity compared to rGelonin. Thus, designing a new fusion construct should be performed. This may include inserting a flexible linker between the scFvCD133 gene and the gene coding for gelonin to relieve steric hindrance of the enzymatic RIP activity of the immunotoxin.

The CD133 receptor specificity of scFvCD133/rGelonin was demonstrated in vitro by the increased accumulation of scFvCD133/rGelonin on the plasma membrane of CD133^high^ HT-29 cancer cells, while not on the U87 cells, which have high intracellular expression of CD133 [42]. This was also verified by flow cytometry (Supplementary Figure S2). Another observation that support the specificity of the scFvCD133/rGelonin is that the only cell line that did not have any drop in viability after 72 h incubation of 333 nM scFvCD133/rGelonin (or rGelonin) and no difference of cytotoxic effects between PCI of scFvCD133/rGel and PCI of rGelonin was the NIH/3T3 cells, which has been shown by others to be CD133 negative, both on the mRNA [43] and the protein level [35]. However, as for the WiDr and HT-29 cells, we could not separate the PDT effect from the PCI of rGelonin effect in the NIH-3T3 cells. We can therefore not exclude that other parameters affecting sensitivity are also involved. Of relevance, Swaminathan et al. demonstrated CD133-selectivity of scFvCD133, which is derived from the mAb clone CD133 #HB7 (clone 7), and the very same scFv used in this study: scFvCD133 did not bind to NIH/3T3 wild-type cells, but did bind to NIH/3T3 cells transfected with the mouse CD133 gene [35]. We decided to use the NIH/3T3 cell line as a CD133 negative control due to the following reasons: (1) The NIH/3T3 cells do not express the mRNA for CD133 [43]; (2) The NIH/3T3 cell line has previously been used as control cells for scFv-based CD133-targeting using the very same scFv (anti-CD133 hybridoma clone 7) as used in this study [35]; (3) As the NIH/3T3 cells are immortalized they cannot be defined as normal fibroblast, however, they are commonly used as a standard fibroblast cell line; (4) The scFvCD133/rGelonin immunotoxin is designed to target not only human CD133 but also mouse CD133 (prominin-1) [35], which will allow us to assess PCI of the immunotoxin in immunocompetent mice and potential adverse effects.; (5) The PCI technology has been, and currently is, under evaluation in clinical trials. The tumors, and their surrounding normal tissues, treated with PCI have most truly been containing fibroblast cells. These trials, with their official titles, include: (a) “Phase I, Dose-escalating Study to Evaluate Safety and Tolerance of Amphinex Based Photochemical Internalization (PCI) of Bleomycin in Patients With Local Recurrence or Advanced/Metastatic, Cutaneous or Sub-cutaneous Malignancies” (ClinicalTrials.gov Identifier: NCT00993512) [44]; (b) “A Phase I/II Dose Escalation Study to Assess the Safety, Tolerability and Efficacy of Amphinex^®^-Induced Photochemical Internalization (PCI) of Gemcitabine in Patients With Advanced Inoperable Cholangiocarcinomas” (ClinicalTrials.gov Identifier: NCT01900158); and (c) “An Open-label, Phase I/Proof of Principle, Dose Escalation Study to Assess Safety, Tolerability and Immune Response of Fimaporfin-induced Photochemical Internalization (PCI) of Antigen/Adjuvant in Healthy Male/Female Subjects” (ClinicalTrials.gov Identifier: NCT02947854). Therefore, based on these rationales, we conclude that the mouse cell line NIH/3T3 is a valid negative control for evaluating the specificity of scFVCD133/rGelonin, both alone and in combination with the PCI method. Furthermore, the intracellular accumulation of scFvCD133/rGelonin was also found increased compared to that of rGelonin after 24 h incubation in HT29 CD133^high^ cells (data not shown). The fluorescence puncta observed after 24 h incubation of scFvCD133/rGelonin, indicate that the immunotoxin has been entrapped in endosomes and/or lysosomes, which is in line with previous work of Stratford et al. and Bostad et al. demonstrating the co-localization of CD133 targeting immunotoxins and TPCS_2a_ with LysoTracker [10,33]. CD133 stability has been shown to be regulated by HDAC6 through the endo-/lysosomal pathway [45]. In our study, TPCS_2a_-PCI dramatically enhanced the cytotoxic potential of scFvCD133/rGelonin indirectly demonstrating that scFvCD133/rGelonin co-localizes with the endo-/lysosomal localizing PCI-photosensitizer TPCS_2a_. Despite a three orders of magnitude attenuation of N-glycosidase/RIP activity of scFvCD133/rGelonin compared to rGelonin, PCI of scFvCD133/rGelonin induced superior cytotoxic responses than PCI of rGelonin, indicating an efficient receptor-mediated endocytosis of the CD133-targeting immunotoxin. In addition, even though scFvCD133/rGelonin exhibits specificity for binding and accumulation in CD133^high^ cells, PCI of scFvCD133/rGelonin also induced higher toxicity than PCI of rGelonin in CD133^low^ (U87, MDA-MB-231) cell lines. Of relevance for this observation, it has been estimated that only 1–10 molecules of RIP toxins (gelonin) is sufficient to kill a cell [46], suggesting that cells with low expression of CD133 will be sensitive to PCI of scFvCD133/rGelonin. Thus, the low intracellular accumulation of scFvCD133/rGelonin in the CD133^low^ U87 cell line (Figure 2b) is therefore sufficient for PCI-mediated cell death. Of relevance to this observation, high intracellular expression of CD133 has been detected in U87 glioma cells [42]. Based on this, and a recent study by Izumi et al. showing that small fractions of CD133 is transported out to the plasma membrane via recycling endosomes in the U87 cells [47], we suggest that this provides sufficient CD133 receptor-mediated uptake of scFvCD133/rGelonin and reduced viability following PCI of scFvCD133/rGelonin compared to PCI of rGelonin. Concerning the MDA-MB-231 cells, several reports have shown that this cell line is also CD133-positive (as in this study, mAb clone 7 was used) [48,49]. Cellular uptake of rGelonin occurs by pinocytosis [50]. Thus, the lack of fluorescence signals from rGelonin both in the HT29 cells and the U87 cells (Figure 2b), indicate that the fluorescence puncta of scFvCD133/rGelonin in the U87 cells, observed after 1 h incubation, is due to efficient internalization by receptor-mediated endocytosis of the immunotoxin.

To further assess the efficacy and specificity of svFvCD13/rGelonin, we FACSorted the 5% highest and 5% lowest CD133 expressing HT29 cells and assessed these two populations for cytotoxic responses after PCI. Strikingly, PCI of scFvCD133/rGelonin induced strong cytotoxic effects (30-fold increase compared to PCI of rGelonin (*p* = 0.000798) or PDT alone, at 1 min light dose) in the 5% top CD133 population, which is in line with the effects seen in the WiDr cells. In the 5% lowest CD133 expressing HT29 cells, an 8-fold difference in cytotoxicity was detected between cells treated with PCI of scFvCD133/rGelonin and PCI of rGelonin/PDT (*p* = 0.00235). A possible explanation of the relative high cytotoxic effect induced by PCI of scFvCD133/rGelonin in the FACSorted CD133^low^ cells could be due to re-expression of CD133 on the plasma membrane during over-night incubation of the immunotoxin, or simply that a very low expression (as for U87 cells) is sufficient for PCI-induced targeting of CD133. Indeed, we have experimental data showing that FACSorted CD133^low^ WiDr cells over time re-express the CD133 receptor on the plasma membrane (unpublished), which is in agreement with Peickert et al. that showed that CD133^low^ HT-29 and SW620 colorectal adenocarcinoma cells quickly re-express CD133 on their surface both in vitro and in vivo [51]. Despite the very low RIP-activity of scFvCD133/rGelonin compared to free rGelonin, PCI of scFvCD133/rGelonin was found highly effective, which is in line with our previous works on PCI of immunotoxins targeting CD133 based on either whole mAbs (AC133, MW: 150 kDa) conjugated to the RIP saporin (MW: 210 kDa) or linked via AC133-biotin–streptavidin-saporin (MW: >710 kDa) [10,32,33].

CD133 binds to cholesterol in the plasma membrane [52,53], and its function is believed to involve the organization of cell membrane topology [54]. Amino acids, fatty acids and cholesterol are the main targets of singlet oxygen generated by the photochemical reactions from PCI. We therefore suggest that photochemical damage to cholesterol and the location of CD133 in cholesterol-rich areas yield a synergistic effect to the PCI-induced release of scFvCD133/rGelonin. This may explain why PCI of scFvCD133/rGelonin induces a strong cytotoxic response compared to PCI of rGelonin even when only low amounts of the CD133 receptor are present.

One weakness of this study is that we only used the immortalized mouse fibroblast cell line NIH-3T3 cell line as a negative control. Although the scFVCD133/rGelonin immunotoxin is targeting both mouse and human CD133 it is necessary to include a human cell line that is CD133 negative before further pre-clinical evaluations of this targeting strategy. A potential problem regarding CD133-negative cells and their likelihood of survival as the immunotoxin is targeting malignant cells expressing CD133 at various degrees. However, we suggest that these cells will be killed directly (apoptosis or necrosis) or indirectly (vascular shut down or by immunization) by the PDT effect of the therapy [25].

In conclusion, we have developed a recombinant CD133-targeting immunotoxin, named scFvCD133/rGelonin that exerts very low cytotoxic effects alone in CD133-expressing cells. scFvCD133/rGelonin binds to and accumulates in CD133^high^ cells and to a very low degree in CD133^low^ cells. scFvCD133/rGelonin becomes highly cytotoxic compared to non-targeted rGelonin when only activated by the light-controlled, intracellular drug delivery method PCI in both CD133^high^ and CD133^low^ cells. Further optimization of scFvCD133/rGelonin and preclinical evaluations in combination with the PCI technology is warranted.

## Supplementary Materials

The following are available online at https://www.mdpi.com/2077-0383/9/1/68/s1, Figure S1: Binding and internalization of anti-CD133 mAb, Figure S2: Accumulation of rGelonin and scFvCD133/rGelonin in HT29 and U87 cells.
