# Supplementary material for: Design, Characterization, and Evaluation of scFvCD133/rGelonin: A CD133-Targeting Recombinant Immunotoxin for Use in Combination with Photochemical Internalization
Source: J Clin Med. 2019 Dec 26;9(1):68. doi: 10.3390/jcm9010068 (PMC7019722; doi:10.3390/jcm9010068)
Supplement: Supplementary file 1 [file jcm-09-00068-s001.pdf]

Journal of
Clinical Medicine

Supplementary Materials

Design, characterization and evaluation of scFvCD133/rGelonin: A CD133-targeting recombinant immunotoxin for use in combination with photochemical internalization

Cathrine Elisabeth Olsen[1], Lawrence H. Cheung[2], Anette Weyergang[1], Kristian Berg[1], Daniel A. Vallera[3], Michael G. Rosenblum[4], Pål Kristian Selbo[1,*]

1 Department of Radiation Biology, Institute for Cancer Research, Oslo University Hospital, The Norwegian Radium Hospital, Oslo, Norway;
2 Department of Experimental Therapeutics, The University of Texas MD Anderson Cancer Center, Houston, Texas, USA;
3 Department of Therapeutic Radiology-Radiation Oncology, University of Minnesota, Masonic Cancer Center, Minneapolis, MN, USA;
* Correspondence: selbo@rr-research.no; Tel.: +47-22781469

Figure S1

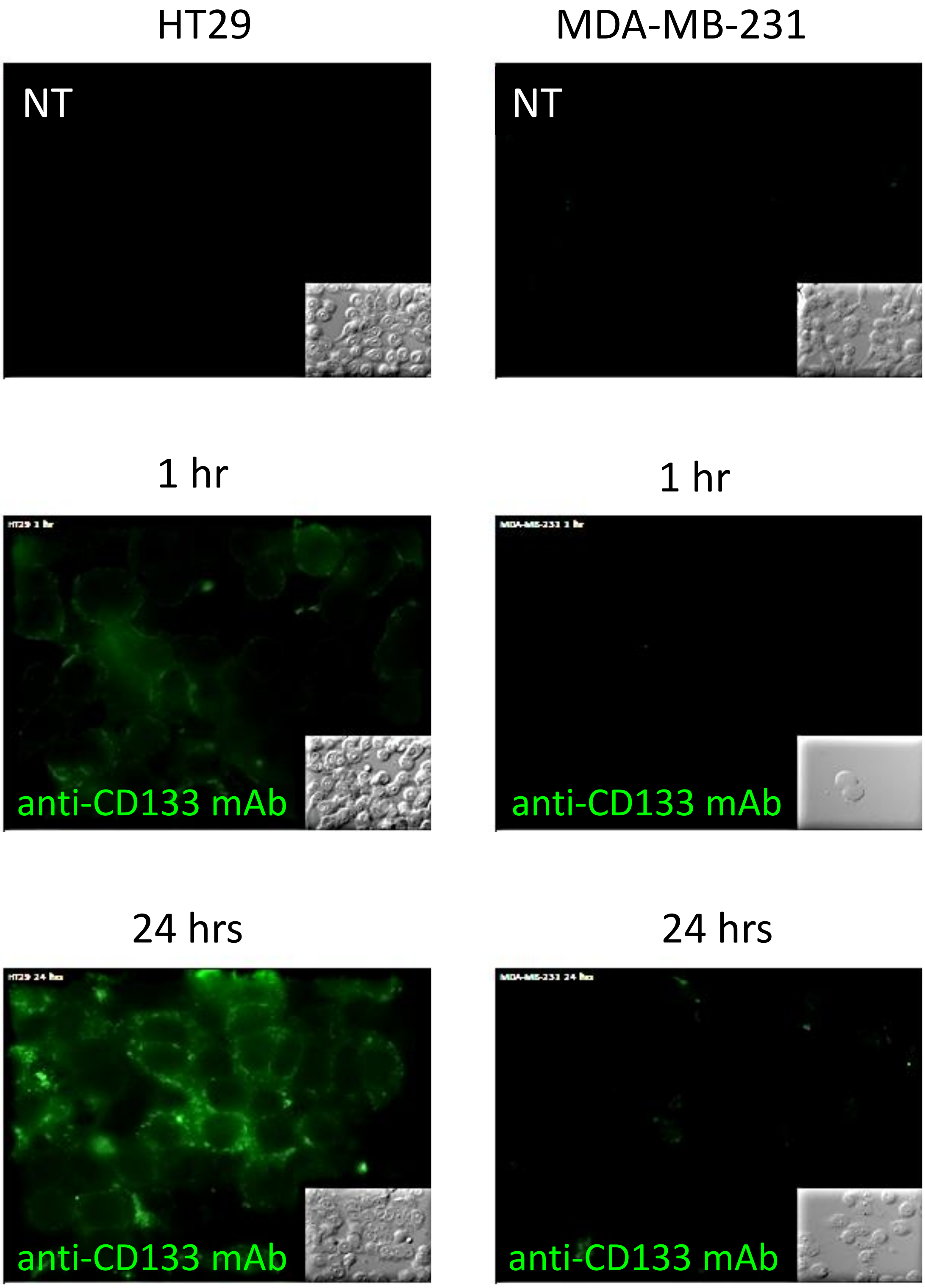

Figure S1. Binding and internalization of anti-CD133 mAb (HB#7). 1 and 24 hrs after incubation of anti-CD133 mAb HB#7, the binding and internalization of HB#7 was evaluated by fluorescence microscopy of HT29 (CD133^{high}) and MDA-MB-231 (CD133^{low}) cancer cells. Inserted gray-scale panels are corresponding DIC microscopy images. The results are representative of three individual experiments.

Suppl.Figure y.

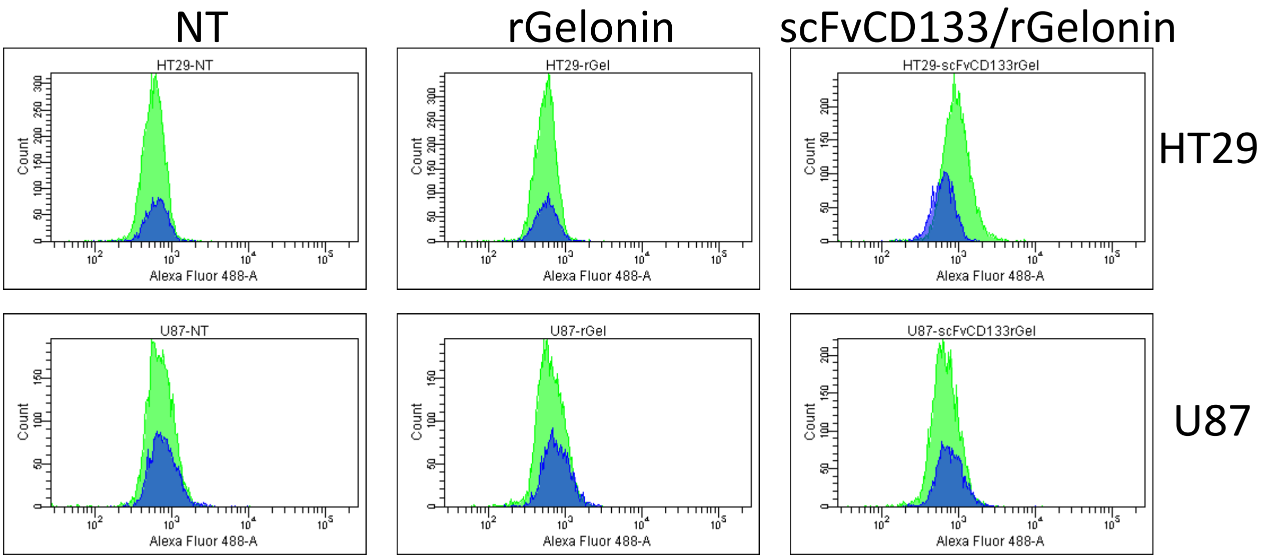

Figure S2. Accumulation of 10 nM rGelonin and 10 nM scFvCD133/rGelonin detected with anti-rGelonin polyclonal Ab and anti-rabbit-Alexa488 mAb (green) in HT29 and U87 cells post 24 hrs incubation, addressed by flow cytometry. The results are representative from two individual experiments, and are included internal untreated controls (barcoded using CellTraceViolet) shown in blue.
